# Design of LabVIEW®-based software for the control of sequential injection analysis instrumentation for the determination of morphine

**DOI:** 10.1155/S1463924602000135

**Published:** 2002

**Authors:** Claire E. Lenehan, Neil W. Barnett, Simon W. Lewis

**Affiliations:** Centre for Chiral and Molecular Technologies School of Biological and Chemical Sciences Deakin University Geelong 3217 Australia

## Abstract

LabVIEW^®^-based software for the automation of a sequential injection analysis instrument for the determination of morphine is presented. Detection was based on its chemiluminescence reaction with acidic potassium permanganate in the presence of sodium polyphosphate. The calibration function approximated linearity (range 5 × 10^-10^ to 5 × 10^-6^ M) with a line of best fit of y=1.05_x_+8.9164 (R^2^ =0.9959), where y is the log10 signal (mV) and x is the log10 morphine concentration (M). Precision, as measured by relative standard deviation, was 0.7% for five replicate analyses of morphine standard (5 × 10^-8^ M). The limit of detection (3σ) was determined as 5 × 10^-11^  M morphine.


Journal of Automated Methods & Management in Chemistry
Vol. 24, No. 4 (July–August 2002) pp. 99–103

Taylor & Francis

Taylor & Francis Group

### Design of LabVIEW®-based software for the control of sequential injection analysis instrumentation for the determination of morphine

**Claire E. Lenehan, Neil W. Barnett* and Simon W. Lewis**
*Centre for Chiral and Molecular Technologies, School of Biological and Chemical Sciences, Deakin University, Geelong, Australia 3217*

*LabVIEW®-based software for the automation of a sequential injection analysis instrument for the determination of morphine is presented. Detection was based on its chemiluminescence reaction with acidic potassium permanganate in the presence of sodium polyphosphate. The calibration function approximated linearity (range $5 \times 10^{-10}$ to $5 \times 10^{-6}$ M) with a line of best fit of $y = 1.05x + 8.9164$ ($R^2 = 0.9959$), where y is the $\log_{10}$ signal (mV) and x is the $\log_{10}$ morphine concentration (M). Precision, as measured by relative standard deviation, was 0.7% for five replicate analyses of morphine standard ($5 \times 10^{-8}$ M). The limit of detection (3σ) was determined as $5 \times 10^{-11}$ M morphine.*

### Introduction

Optimization and control of modern chemical processes requires high-quality chemical information [1, 2]. Such information is ideally provided in real time using process analytical chemistry. Flow injection analysis (FIA) is a powerful, well-established, sample-handling technique well suited to this type of chemistry and it has been applied to online process analysis in industrial [1], fermentation [3] and environmental [4, 5] analysis. The application of FIA to online process analysis can be hampered by the requirement for complex manifolds. In addition, peristaltic pump tubing is generally not compatible with harsh sample matrices [6]. To overcome these limitations, Ruzicka and Marshall introduced sequential injection analysis (SIA) [7]. In contrast to FIA, SIA uses computer-controlled flow programming to afford the application of different chemistries without reconfiguration of the flow manifold [8,9]. SIA manifolds comprise a multiposition valve operating in synchronization with a pump and a suitable detector. The manifold is robust, easily maintained and ideally suited to online process analysis [8]. It is essential that the entire instrument is computer controlled, as precise timing of the pump and valve is required to achieve controlled partial dispersion of the reagent and sample [7,10]. The configuration of SIA instrumentation for the determination of morphine in process streams necessitated the writing of suitable software to control the system and perform data acquisition.

This paper describes the design and application of software written within the National Instruments LabVIEW® graphical programming environment [11–13] to automate fully SIA instrumentation and data acquisition for the determination of morphine in process liquors. The LabVIEW® software facilitated the design of virtual instruments, which allowed synchronized control and data acquisition for the entire analytical system.

### Materials and methods

### *Hardware*

All experiments were performed using a purpose-built sequential injection analysis instrument (figure 1). Control of the pump (Cavro XP-3000, Global FIA, Gig Harbour, Washington, USA) and the 10-port multiposition valve (Valco C25Z, SGE, Melbourne, Australia) was achieved using a desktop computer (Pentium 133 MHz, 32 MByte RAM, Posicom, Geelong, Victoria, Australia) equipped with a data acquisition board (LabPC 1200, National Instruments, Ringwood, Victoria, Australia) running software written in-house using LabVIEW® v.6.0 (National Instruments). Detection was accomplished using a custom-built flow-through chemiluminometer, the details of which are as follows. A glass spiral flow cell (Embell Scientific, Murwillimbah, New South Wales, Australia) was mounted flush against a photomultiplier tube (Thorn EMI Type 9828, ETP Ltd, Ermington, New South Wales, Australia) operating at a constant 800 V supplied by a stable power supply (Thorn EMI Model PM28BN) via a voltage divider supply (Thorn EMI Model C611). All tubing was 0.8 mm i.d. PTFE (ProTECH Pty Ltd, Coolum Beach, Queensland, Australia).

### *Reagents and analytes*

Deionized water and analytical grade reagents were used unless otherwise stated. Stock morphine solutions, working standards and acidic permanganate reagents were all prepared by dissolution in an acidic solution (0.05 M sulphuric acid; Ajax Chemicals, Aurburn, New South Wales, Australia) of sodium polyphosphate (Aldrich, Castle Hill, New South Wales, Australia). Stock morphine solutions were prepared by dissolution of the free base (Glaxo Smith Kline, Port Fairy, Victoria, Australia)

*To whom correspondence should be addressed. e-mail: barnie@deakin.edu.au

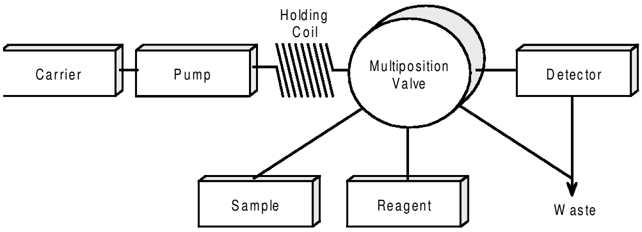

*Figure 1. SIA manifold for the determination of morphine in process samples using acidic potassium permanganate chemiluminescence detection.*

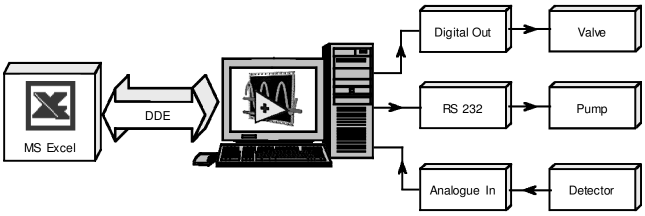

*Figure 2. Computer–SIA connections.*

with working standards prepared by serial dilution. Acidic permanganate solutions were prepared by dissolution of potassium permanganate (Merck Ltd, Poole, UK).

### Results and discussion

### *Software design*

The basic layout and requirements of the SIA system are shown in figure 2. The syringe pump was controlled through the RS 232 serial port whilst the multiposition valve was manipulated using digital-out signals from the data-acquisition board. The board also allowed data collection using differential input from two analogue-input channels. Virtual instruments, developed within LabVIEW®, consist of a user interface and a graphical data flow diagram that contains the source code. These are modular and hierarchical and, as such, can be used as stand-alone programs or as a subprogram (subvirtual instrument). Using this facility, individual virtual instruments for the control of each component and for data acquisition were developed, tested and then linked together to form the final program.

A virtual instrument module was designed for the electronic actuation of the multiposition valve using TTL high/low signals on a single digital out-control line. The module could 'step' the valve to the next port, send the valve to the 'home' position and 'reset' the valve. Control of the syringe pump was achieved using a second virtual

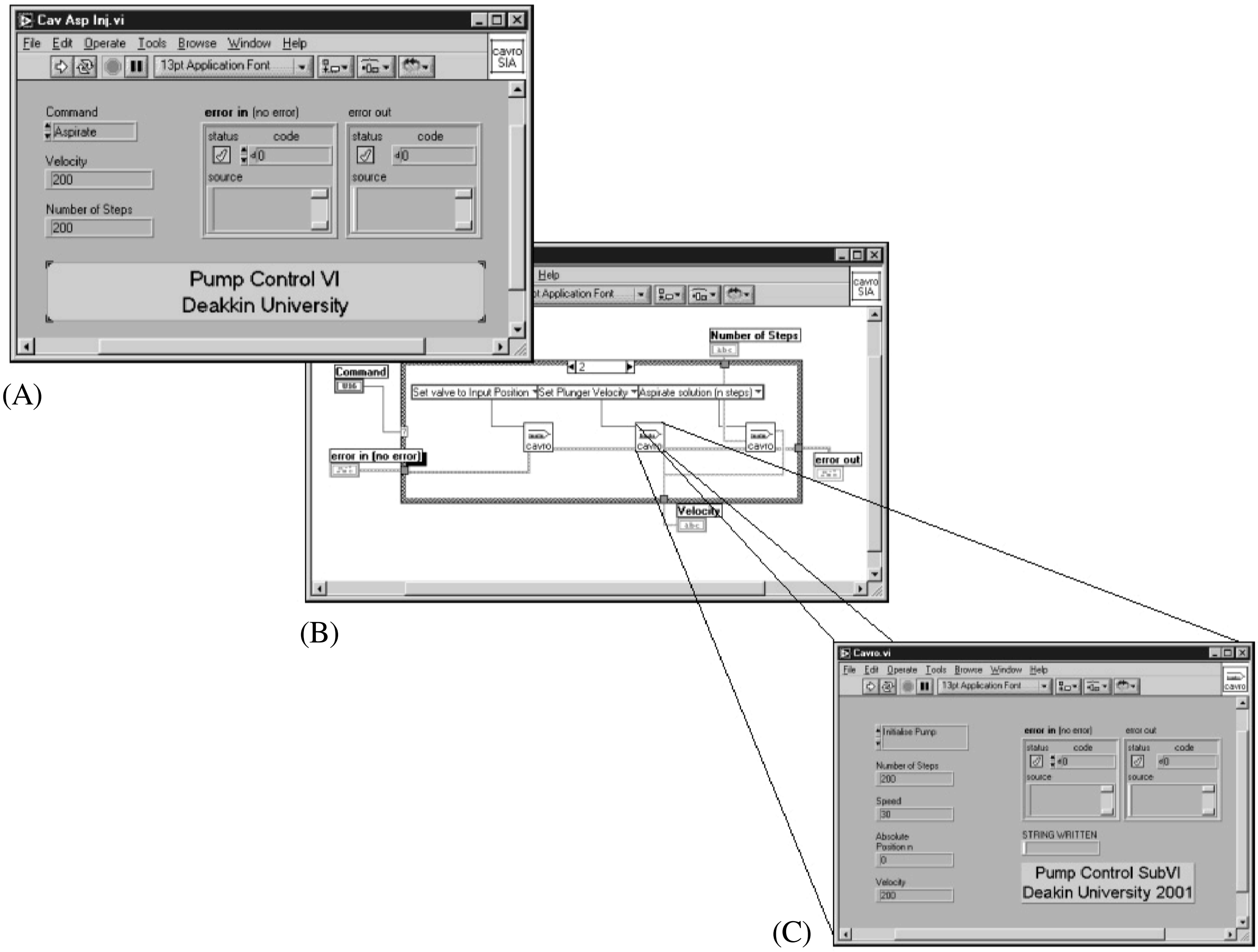

*Figure 3. Structural levels of the syringe pump virtual instrument. (A) Front panel of the virtual instrument showing the pump controls; (B) virtual instrument diagram showing the input terminals wired to an icon representing a subvirtual instrument that this program is calling; (C) front panel of the subvirtual instrument that is being called.*

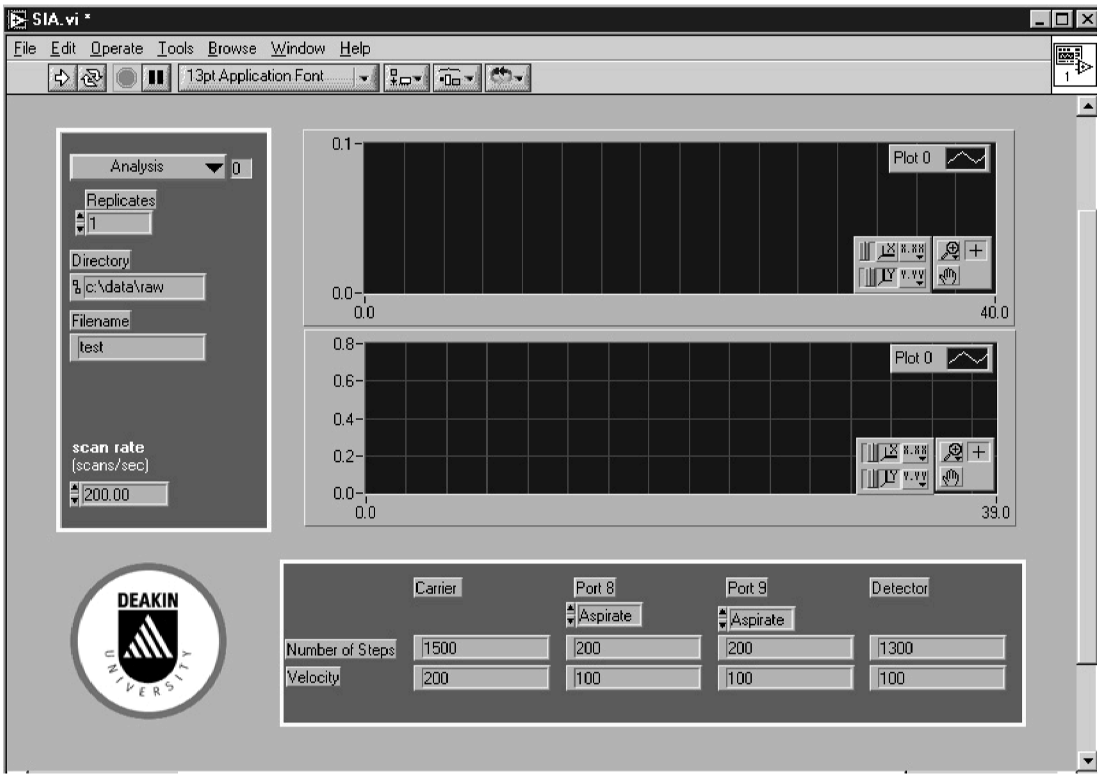

*Figure 4. Front panel of the sequential injection software. On the left side of the display are the data storage options, including file name, directory and the sampling rate. At the bottom of the display are the pump controls; variables include the number of replicates, the direction of the syringe pump, the solution volume and flow rate. The user can also input whether the entire routine is an initialization procedure, loading the pump or analysis. The graphical display shows the most recently acquired data and autoscales on both axes.*

instrument; commands written in ASCII format were sent from the computer to the pump via an RS-232 connection (figure 3). The front panel (figure 3A) shows three user inputs: pump flow rate (velocity), direction (command) and volume (number of steps). The corresponding flow diagram (figure 3B) shows the source code; user inputs are wired to icons that represent a subprogram that the virtual instrument is executing with its front panel shown in figure 3C.

A third virtual instrument was designed to acquire and process analogue data from the photomultiplier tube. This module displays graphically both raw and smoothed data and allows the user to choose the data-acquisition rate. The sampling period is calculated by the software and is dependent upon the flow rate and injection volume. The high and low limit settings for the input signals allowed accurate digital reproduction of the SIA detector response profile. The acquired data were digitally filtered (Butterworth filter within LabVIEW®) and saved in either ASCII or Microsoft Excel formats with dynamic data exchange facilitating the latter.

The front panel of the SIA virtual instrument is shown in figure 4 with the data file-saving options, pump controls and graphical display positioned on the left, bottom and right of the screen, respectively. A selector button in the top left-hand corner gives the user control over the type of experiment to be conducted (either programme initialization, loading solutions or analysis). The virtual instrument hierarchy used to control the instrument is shown in figure 5. For a free copy of the executable software, contact the authors.

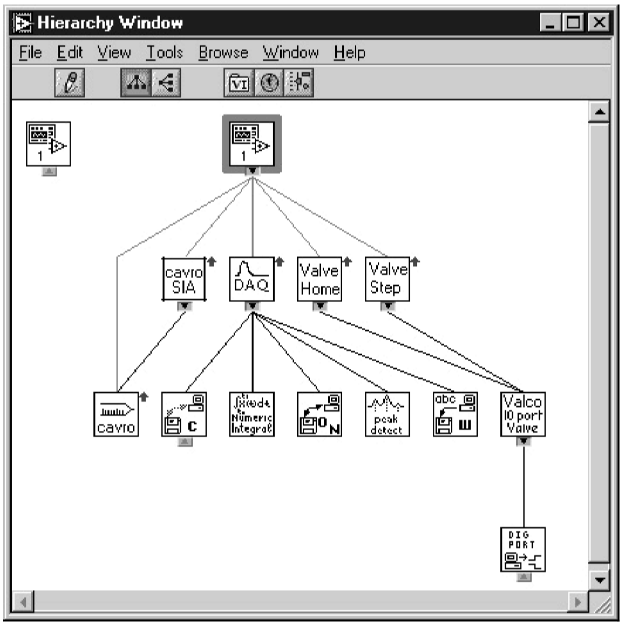

*Figure 5. Virtual instrument hierarchy.*

### *Determination of morphine*

Flow-injection analysis determination of morphine based on its chemiluminescence reaction with acidic potassium permanganate in the presence of polyphosphates was first reported in 1986 [14]. This chemistry was adapted for the

*Table 1.*

| | Previous approach [16] | Present approach |
|---|---|---|
| Calibration function | $y = 1.0 \times 10^{15}x^3 - 2.2 \times 10^{11}x^2 + 1.3 \times 10^7 x - 8.3$ | $y = 1.05x + 8.9164$ ($R^2 = 0.9959$) |
| Linear calibration range | $2.5 \times 10^{-6}$ M–$3.0 \times 10^{-5}$ M | $5 \times 10^{-10}$ M–$5 \times 10^{-6}$ M |
| Limit of detection | $1 \times 10^{-8}$ M | $5 \times 10^{-11}$ M |
| Precision (%rsd) | 1.4% ($n = 15$) at $1.25 \times 10^{-5}$ M | 0.7% ($n = 5$) at $5 \times 10^{-8}$ M |

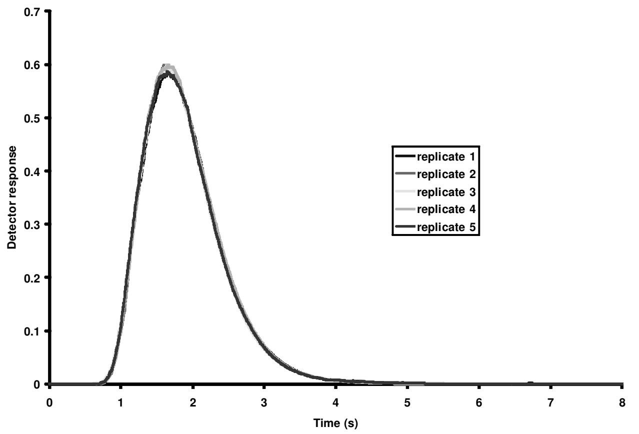

*Figure 6. Five replicate injections of a $6.0 \times 10^{-6}$ M morphine standard, overlaid.*

determination of morphine in process samples in 1993 [15] and more recently was modified to suit SIA for both aqueous and non-aqueous process extracts [16,17]. Consequently, we used this well-established detection chemistry to test the performance of the present automated system.

Potassium permanganate ($5.0 \times 10^{-4}$ M) and morphine standards were prepared in sodium polyphosphate (1.0% m/v) and adjusted to pH 2.0 with sulphuric acid. The carrier solution was sodium polyphosphate (1.0% m/v) in sulphuric acid (pH 2.0). The three-way valve on the pump was set to the left, and carrier solution (3.1 ml) was drawn into the syringe at 12 ml min$^{-1}$. The three-way valve was then set to the right and port 8 chosen on the multipositon valve. Potassium permanganate reagent (250 μl) was aspirated into the holding coil at 1.2 ml min$^{-1}$, the multiposition valve was switched to port 9 and sample (50 μl) was aspirated into the holding coil at 1.2 ml min$^{-1}$. The multiposition valve was then switched to port 10 and the entire volume (3.4 ml) was flushed passed the detector at 12 ml min$^{-1}$.

The analytical figures of merit obtained with this system are superior to those reported previously [16] (table 1). The instrumental reproducibility is demonstrated in figure 6, which shows five replicate injections ($6.0 \times 10^{-6}$ M) overlaid. The detection limit of $5.0 \times 10^{-11}$ M is, to the best of our knowledge, the lowest achieved for morphine using this chemistry. The lower detection limit achieved with the current system can be attributed to the replacement of the peristaltic pump [16] by a syringe pump and significant improvements in the 'light tightness' of the instrument housing as both these changes improved the signal-to-noise ratio. The high precision (table 1) can be attributed to the superior hydrodynamic control attainable with a syringe pump, as the volumes delivered and flow rates are not susceptible to variation through changes in pump tubing dimensions. This instrumentation and chemistry is presently being adapted for the determination of morphine and other related alkaloids in process samples with results to be published in due course.

### Conclusions

The complete automation of an SIA instrument was achieved using software written with LabVIEW®. Individual modules were written for each component and linked to form the final program. The instrumentation was applied to the determination of morphine, with significant improvements to the analytical figures of merit reported previously [16].

### Acknowledgements

The authors thank Dr Richard Bos, Deakin University, for assistance with building the lightproof instrument housing.

### References

1. Beebe, K. R., Blaser, W. W., Bredeweg, R. A., Chauvel, J. P., Harner, R. S., LaPack, M., Leugers, A., Martin, D. P., Wright, L. G. and Yalvac, E. D., *Analytical Chemistry*, **65** (1993), 199R.
2. Hassell, D. and Bowman, E. M., *Applied Spectroscopy*, **52** (1998), 18A.
3. Christensen, L. H., Marcher, J., Schulze, U., Carlsen, M., Min, R. W., Nielsen, J. and Villadsen, J., *Biotechnology and Bioengineering*, **52** (1996), 237.
4. Reis, B. F., Morales-Rubio, A. and Guardia, M. D. L., *Analytica Chimica Acta*, **392** (1999), 265.
5. Andrew, K. A., Blundell, N. J., Price, D. and Worsfold, P. J., *Analytical Chemistry*, **66** (1994), 916A.
6. Martinez Calatayud, J., *Flow Injection Analysis of Pharmaceuticals: Automation in the Laboratory* (London: Taylor & Francis, 1996).
7. Ruzicka, J. and Marshall, G. D., *Analytica Chimica Acta*, **237** (1990), 329.
8. Barnett, N.W., Lenehan, C. E. and Lewis, S.W., *TrAC — Trends in Analytical Chemistry*, **18** (1999), 346.
9. Ruzicka, J. and Hansen, E. H., *TrAC — Trends in Analytical Chemistry*, **17** (1998), 69.
10. Marshall, G. D. and van Staden, J. F., *Analytical Instrumentation*, **20** (1992), 79.

11. LabVIEW v.6.0 (Austin: National Instruments, 2000).
12. De Viteri, F. J. S. and Diamond, D., *Analytical Proceedings*, **31** (1994), 229.
13. Powell, M. and Tempst, P., *Analytical Chemistry*, **73** (2001), 776.
14. Abbott, R. W., Townshend, A. and Gill, R., *Analyst*, **111** (1986), 635.
15. Barnett, N. W., Rolfe, D. G., Bowser, T. A. and Paton, T. W., *Analytica Chimica Acta*, **282** (1993), 551.
16. Barnett, N. W., Lewis, S. W. and Tucker, D. J., *Fresenius Journal of Analytical Chemistry*, **355** (1996), 591.
17. Barnett, N. W., Lenehan, C. E., Lewis, S. W., Tucker, D. J. and Essery, K. M., *Analyst*, **123** (1998), 601.

